# The Calculator of Anti-Alzheimer’s Diet. Macronutrients

**DOI:** 10.1371/journal.pone.0168385

**Published:** 2016-12-19

**Authors:** Marcin Studnicki, Grażyna Woźniak, Dariusz Stępkowski

**Affiliations:** 1 Department of Experimental Design and Bioinformatics, Warsaw University of Life Sciences-SGGW, Warszawa, Poland; 2 Laboratory of Molecular Basis of Cell Motility, Nencki Institute of Experimental Biology, Warszawa, Poland; Nathan S Kline Institute, UNITED STATES

## Abstract

The opinions about optimal proportions of macronutrients in a healthy diet have changed significantly over the last century. At the same time nutritional sciences failed to provide strong evidence backing up any of the variety of views on macronutrient proportions. Herein we present an idea how these proportions can be calculated to find an optimal balance of macronutrients with respect to prevention of Alzheimer’s Disease (AD) and dementia. These calculations are based on our published observation that per capita personal income (PCPI) in the USA correlates with age-adjusted death rates for AD (AADR). We have previously reported that PCPI through the period 1925–2005 correlated with AADR in 2005 in a remarkable, statistically significant oscillatory manner, as shown by changes in the correlation coefficient R (R_original_). A question thus arises what caused the oscillatory behavior of R_original_? What historical events in the life of 2005 AD victims had shaped their future with AD? Looking for the answers we found that, considering changes in the per capita availability of macronutrients in the USA in the period 1929–2005, we can mathematically explain the variability of R_original_ for each quarter of a human life. On the basis of multiple regression of R_original_ with regard to the availability of three macronutrients: carbohydrates, total fat, and protein, with or without alcohol, we propose seven equations (referred to as “the calculator” throughout the text) which allow calculating optimal changes in the proportions of macronutrients to reduce the risk of AD for each age group: *youth*, *early middle age*, *late middle age and late age*. The results obtained with the use of “the calculator” are grouped in a table (Table 4) of macronutrient proportions optimal for reducing the risk of AD in each age group through minimizing Rpredicted−i.e., minimizing the strength of correlation between PCPI and future AADR.

## Introduction

The influence of diet on human organism is very complex and should be considered in its totality. It is trivial to say that overconsumption or under consumption of certain nutrients or food types may lead to delayed health consequences. It is becoming more and more recognized that consumption of macronutrients in optimal *proportions* has stronger beneficial health effects than consumption of the optimal *amount* of a particular macronutrient [1–5].

The state of the art studies in nutritional sciences assume that we should provide all the necessary nutrients in appropriate amounts throughout the life to assure proper development, functioning and healthy aging. Searching for the appropriate amounts of dietary intake remains the main aim of nutritional studies. Traditional epidemiological approach used to study the relation between macronutrient content of a diet and the development of chronic diseases is based on the analysis of food intake questionnaires recalculated to estimate individual macronutrient intake and dietary proportion of particular macronutrients. In the majority of such studies the population is divided into quartiles or quintiles according to the amount of the consumed macronutrient. Such distribution data are then usually used to build a proportional hazard model (Cox model) allowing for calculation of hazard ratios of disease onset for each quartile or quintile of macronutrient consumption. Unfortunately, there is only one report pertinent to the relation between macronutrients consumption and mild cognitive impairment (MCI) and dementia risk [6]. This report point to negative influence of high carbohydrates consumption but beneficial influence of high fat and protein consumption. In a more general approach Wang et al. [7] reported hazard ratios for mortality from neurodegenerative diseases, not only MCI and dementia, in relation to consumption of fat and its various components [7]. This [7] and another report [8] are dealing with macronutrients and all-cause mortality. Considering maintaining health and longevity, these reports point to the apparent risk of high carbohydrates consumption but beneficial effects of high total fat intake. As to the types of fat—trans and saturated fats are increasing the risk [7] while mono and polyunsaturated fats are beneficial [7,8]. Of note, Solfrizzi et al. [8] observed that the ratio between pooled unsaturated to saturated fats was positively related to the risk of all-cause mortality. This issue needs further clarification.

Herein we present a novel statistical model that allows calculating macronutrient proportions that have the strongest prophylactic effect when it comes to AD development. We quantified those proportions by analyzing the susceptibility of the American population to Alzheimer’s disease, together with historical macronutrient availability data in the U.S.A. We found that, consistent with other available literature data, every period of human life (youth, early middle age, late middle age, late age) has its own optimal proportion of “anti- AD” macronutrient intake.

Since there is no cure for AD the nutritional approach to prevention of this disease on an individual and population scale, seems to be a rational strategy.

## Methods

**Fig 1:**
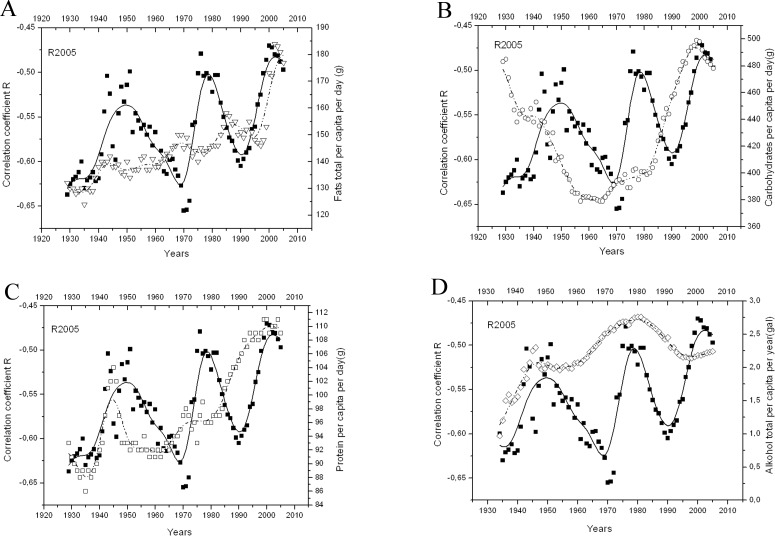
**ABCD** The time course of the availability of four macronutrients and variability of R in the period 1929–2005. A Fat total, B Carbohydrates, C Protein, D Alcohol total.

Typically the relation between varying macronutrient proportions in the diet and health outcomes is studied in small populations by analyzing food questionnaires filled by patients. The percent share of the total energy intake supplied by particular macronutrients can be easily calculated. Here we propose a different strategy—we are looking for the relation between the proportions of macronutrients consumed by the studied population of the U.S.A. in the past and the future susceptibility to Alzheimer’s disease, measured as age-adjusted death rates (AADR). Since we have previously observed that the raw parameter of a lifestyle healthiness (per capita personal income, PCPI) correlates with AADR for AD [9] it seems that relating population statistical data describing social and lifestyle habits to the susceptibility to disease might be a promising strategy. In this paper we try to explain the reason behind the oscillations of correlation strength (measured by R_original_) between PCPI and AD AADR in 2005 that we have observed previously [9]. Since changes in the macronutrient availability in the period 1929–2005 resemble changes of R_original_ in time [9] but usually with time shifts (precedence of peaks) (see Fig 1A, 1B, 1C and 1D), we have attempted to check whether they can explain the variability of R_original_ that we have previously observed.

**Fig 2:**
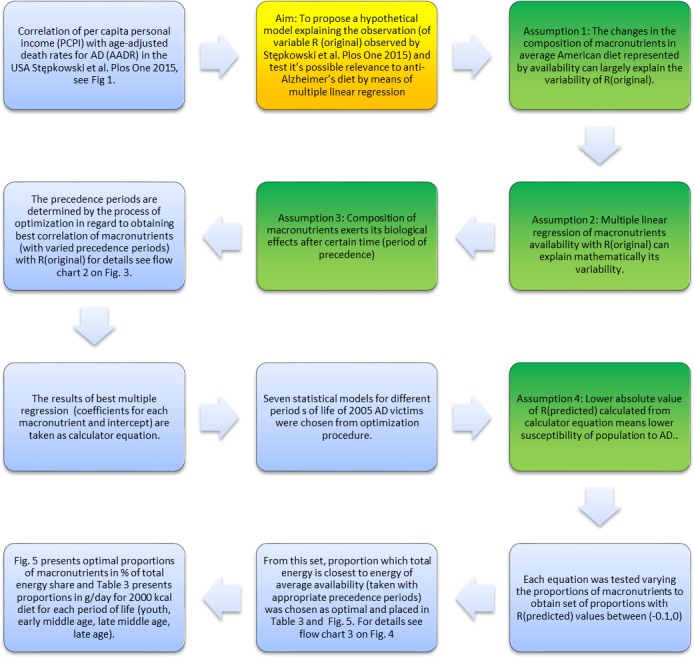
Flow chart 1 of the rationale applied in this paper.

We achieved very high correlations (R ranging from 0.8 to 0.99) by applying optimized precedence periods for the biological consequences of macronutrient intake in the seven studied regression models. Therefore we conclude that, indeed, the variations in R_original_ can be largely explained by variations in macronutrient consumption in the past. Based on the seven regression models studied here we propose a set of optimal prophylactic macronutrient proportions in the diet. The flow chart describing the rationale for this study is presented in Fig 2. The correlation coefficient R_original_ of correlations between PCPI (1925–2005) and AADR 2005 was taken from the previous paper[9]. R_original_ was correlated by a multiple regression procedure implemented in the Statistica package (Statsoft) with macronutrient (carbohydrates, protein, fat total) per capita availability [data from USDA ERS service http://www.ers.usda.gov/data-products/food-availability-%28per-capita%29-data-system/.aspx#26715] and alcohol per capita consumption [10]. The linearization of R oscillations (approximated by 16^th^ degree polynomial) is possible owing to the linear character of the polynomial parameters and replacement of independent variable powers by independent variables of nutrient quantities. The polynomial model of R can be linearized due to the fact that this model is linear in parameters. The general multiple regression equation is as follows y = b0 + b_1_x + b_2_x_2_ + b_3_x_3_ … bpxp. This equation takes a special form when x_1_ = x^1^, x_2_ = x^2^, x_3_ = x^3^ …..x_p_ = x^p^. therefore x_i_ can be replaced by nonlinearly changing nutrient quantities (after Orlov, [11]). The period 1929–2005 was divided into four periods: 1929–1949 *youth*, 1949–1970 *early middle age*, 1970–1990 *late middle age*, 1990–2005 *late age*. This division was based on the assumption that different periods of life require different quantities and proportions of macronutrients. We assume that people who died from AD in 2005 were in majority 70–85 years old. Therefore our division represent**s** four periods of their life. A crucial problem in correlating the consumption of certain nutrients with the disease outcome, in this case the death from AD as the primary cause, is the lag between the cause and the outcome. If we consider bad diet as a cause of disease there has to be a preceding period. It is a case of Granger’s causality [12].

**Fig 3:**
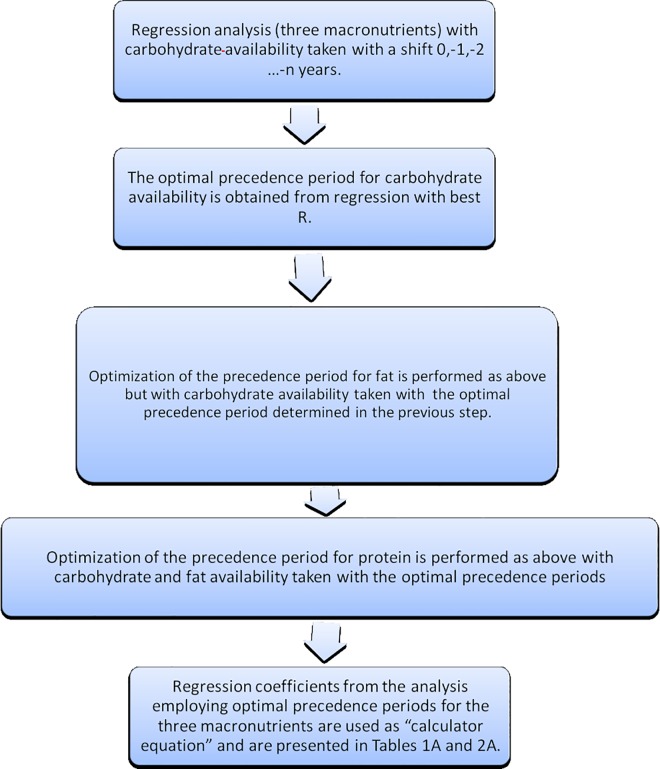
Flow chart 2 optimization of precedence periods.

The examination of temporal changes in macronutrient availability in the period 1929–2005 followed by R_original_ variability brought us to the conclusion that each period and each nutrient has different precedence period in relation to R_original_ variability. The time courses of nutrient availability and R_original_ variability are presented in Fig 1A, 1B, 1C and 1D. To determine the exact period of precedence for the relation between nutrient consumption level and AD susceptibility of the American population, we performed an optimization procedure. This procedure involved multiple linear regressions for all macronutrients taking into account the availability of each nutrient with different precedence periods (in one-year steps). We scanned the time space searching for the highest absolute value of R_optimal_ for each nutrient separately. First we analyzed the carbohydrates to spot the highest R_optimal_ (with respective precedence). Then, with the period of precedence found for carbohydrates, we optimized the second macronutrient and then the third one, with the precedence optimal for the first two macronutrients. In this way we were able to determine the lag period between the biological causes (intake of each nutrient in each period studied) and consequences (changes in the susceptibility to AD measured as death rate) (see flow chart 2 on Fig 3). The regression equations we obtained when searching for optimal precedence periods for all nutrients constitute the set of anti-Alzheimer’s diet calculator equations for *youth*, *early middle age*, *late middle age and late age*. The general form of the calculator equation is as follows:

R = A0 + A1x(nutr 1) + A2x(nutr 2) + Anx(nutr n) where A0..An are coefficients from multiple linear regression (Table 1A and 1B), nutr n–a particular nutrient.

**Table 1:** Regression coefficients for macronutrients without alcohol (A) and with alcohol (B) and their corresponding periods of precedence in years.

A								
Variable	1929–1949		1949–1970		1970–1990		1990–2005	
	Period of precedence	b	Period of precedence	b	Period of precedence	b	Period of precedence	b
Intercept		-1.052		-0.967		-0.489		-1.863
Carbohydrates	10	-0.0007	15	0.0008	1	0.0001	1	0.0017
Fat	0	0.0042	2	-0.0026	10	0.0126	13	0.0069
Protein	5	0.0022	5	0.0043	2	-0.0088	5	-0.001
B								
Variable	1929–1949		1949–1970		1970–1990		1990–2005	
	Period of precedence	b	Period of precedence	b	Period of precedence	b	Period of precedence	b
Intercept				-1.1912		-2.6542		-2.9254
Carbohydrates			13	0.0012	6	0.0031	1	0.003
Fat			2	-0.0027	7	0.0051	11	0.0039
Protein			2	0.0042	0	-0.0039	0	0.0001
Alcohol			0	0.0453	0	0.3678	0	0.2468

These equations can help to determine the macronutrients with the highest influence on the susceptibility to AD and establish their relative proportions in the diet that may turn out to be beneficial for AD prevention.

The tables of macronutrient proportions were prepared by calculating the average nutrient availability in each period and increasing or decreasing the availability by a certain step value from the average level. Carbohydrates and fat total were varied for several fixed protein levels. For the models with alcohol, the alcohol level was fixed at 12.5g or 25 g of pure ethanol daily which correspond to half and one standard drink, respectively. The results are color coded with a yellow background indicating proportions with the minimum R_predicted_ value. The tables, active spreadsheet with the calculator along with manual describing the tables and how to use the spreadsheet, are presented in S1, S2 and the S3 Files.

For each regression model one of the proportions in the yellow range was chosen as optimal for an average person.

**Fig 4:**
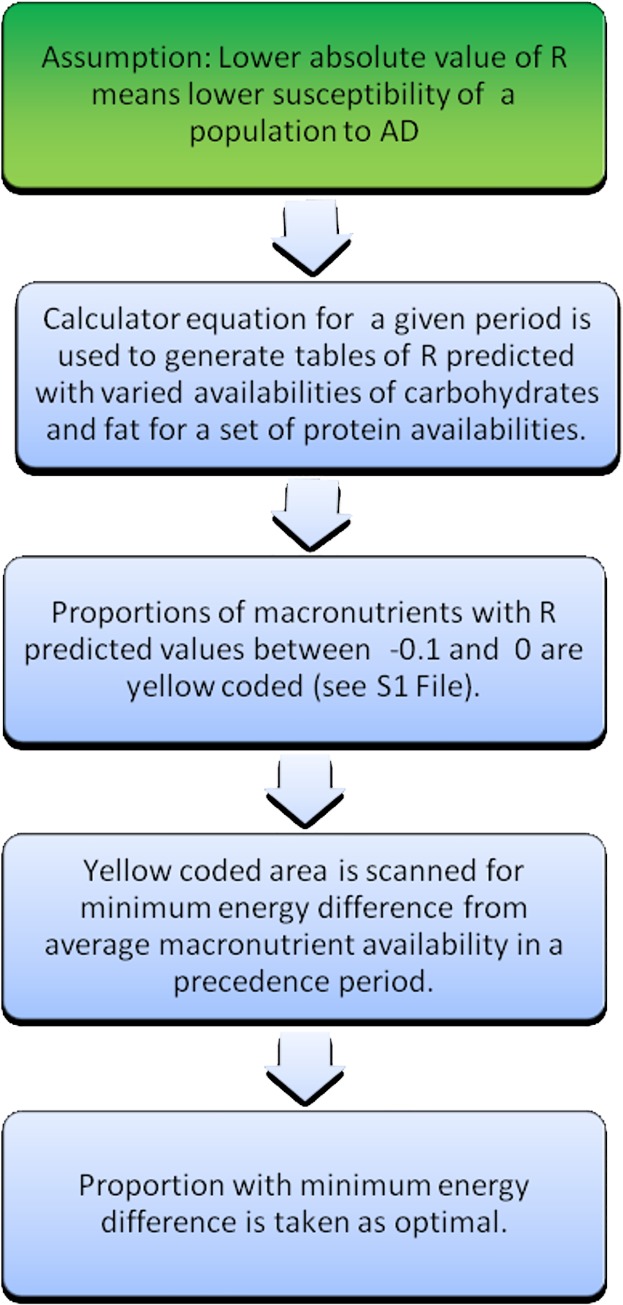
Flow chart 3 optimization of macronutrient proportions.

The choice was made on the basis of the minimum energy difference between the energy derived from macronutrients consumed at the calculated proportions and the energy of macronutrients consumed in proportions related to their availability in each period, taken with precedence periods. For these optimal proportions of macronutrients the percent share of energy of each nutrient and its quantities in g/day, assuming 2000 kcal/day diet, were calculated (see flow chart 3 on Fig 4).

## Results

By applying the procedure of optimization of precedence periods (described in Methods) we have obtained regression models for every period of life: youth, early and late middle age, and late age. Each model was prepared for the three main macronutrients (carbohydrates, fat total and protein) and, with the exception of the model concerning youth, in two variants: with and without the presence of alcohol in the diet. In total, we have obtained seven models listed below:

List of Models

1. Model 1929–1949 *„youth”*
2. Model 1949–1970 *„early middle age”*
3. Model 1949–1970 *„early middle age with alcohol”*
4. Model 1970–1990 *„late middle age”*
5. Model 1970–1990 *„late middle age with alcohol”*
6. Model 1990–2005 *„late age”*
7. Model 1990–2005 *„late age with alcohol”*

Optimization of the preceding period was applied for all models and macronutrients except alcohol for which we used a precedence period of 0 years.

The resulting preceding periods as well as optimal coefficients b from the regression are listed in Table 1A (without alcohol) and 1B (in the presence of alcohol). Table 2A and 2B present either a positive or negative sign of each nutrient coefficient and the impact on the calculated R_predicted_ as the corresponding percent share, indicating which macronutrient has the highest or lowest influence on the calculation results. Table 3A and 3B present the statistical parameters for each model’s optimal (with regard to precedence period) regression. The correlation coefficients R were high and varied from 0,8 to 0,99.

**Table 2:** The values (positive or negative) of macronutrient coefficients and the strength of the influence (in %) of each macronutrient without alcohol (A) and with alcohol (B) in each model. Bold font indicates the nutrient with the highest influence on R_predicted_.

A								
Period	Carbohydrates		Fat		Protein			
	Influence	%	Influence	%	Influence	%		
1929–1949	Negative	29.9	Positive	**49.1**	Positive	20.9		
1949–1970	Positive	**39.7**	Negative	22.8	Positive	37.5		
1970–1990	Positive	2.5	Positive	39.3	Negative	**58.3**		
1990–2005	Positive	43.4	Positive	**45.9**	Negative	10.7		
B								
Period	Carbohydrates		Fat		Protein		Alcohol	
	Influence	%	Influence	%	Influence	%	Influence	%
1929–1949								
1949–1970	Positive	**41.7**	Negative	17.5	Positive	26.8	Positive	14
1970–1990	Positive	28.2	Positive	10.9	Negative	21.1	Positive	**39.8**
1990–2005	Positive	**54.5**	Positive	20.4	Positive	2.4	Positive	22.7

**Table 3:** Parameters of goodness of fit for models without alcohol (A) and with alcohol (B).

A				
Goodness of fit statistics	Period			
	1929–1949	1949–1970	1970–1990	1990–2005
Correlation coefficients R	0.8099	0.9547	0.8026	0.9922
Coefficient of determination R2	0.6559	0.9115	0.6442	0.9844
Adjusted *R*^2^	0.5952	0.8967	0.5815	0.9805
F test	10.8031	61.7822	10.2615	252.9823
p-value	0.0003	0.0001	0.0004	0.0001
Standard error of prediction	0.0412	0.0127	0.0347	0.0069
B				
Goodness of fit statistics	Period			
	1929–1949	1949–1970	1970–1990	1990–2005
Correlation coefficients R		0.9572	0.9343	0.9952
Coefficient of determination R2		0.9161	0.8729	0.9905
Adjusted *R*^2^		0.8964	0.8411	0.987
F test		46.4291	27.475	286.6351
p-value		0.0001	0.0001	0.0002
Standard error of prediction		0.0121	0.0214	0.0056

The listed models were used for predicting the R (R_predicted_) values for each model with a new set of different proportions of nutrients in the diet in order to minimize R_predicted_. For models 1–7 those tables can be found in S1 File. Each table presents a set of two parameters for each proportion of macronutrients. The first parameter is the R_predicted_ value, the second is the value of the total energy of nutrients in a given proportion. The minimum value of the difference from the energy of mean amounts of nutrients (availability) for a given period, taken with the precedence periods, is indicated at the bottom of the table.

The smallest difference in all tables indicates the total energy of macronutrients in a given proportion and in a given period that were closest to the historical values of the total energy of macronutrients calculated from their average availability. We consider the proportion of macronutrients corresponding to this energy as the optimal one–the one most beneficial in AD prevention.

**Fig 5:**
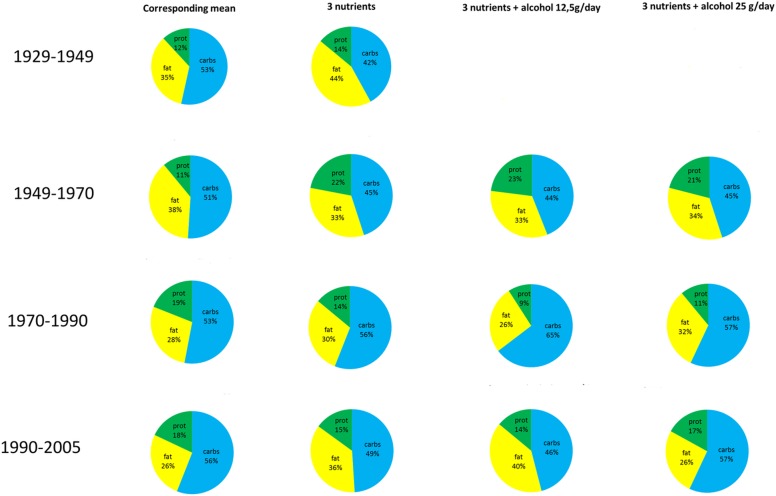
Wheel charts of the proportions of macronutrients in the energy units for each period studied.

Seven proportions corresponding to seven models were calculated to indicate the share of a particular macronutrient in the total energy for a given proportion of macronutrients (see Fig 5) along with gram/day amounts in a 2000 kcal diet (see Table 4). In other words we propose to shift the average macronutrient proportions in food availability and, as its proxy, the average consumption in a certain period of life, according to the results presented in Table 4.

**Table 4:** Comparison of the mean availability of macronutrients in the corresponding periods of precedence with predicted optimal proportions of macronutrients in grams per day assuming 2000 kcal diet. Calculated from models without alcohol and with two levels of alcohol consumption: 12.5 or 25 g pure ethanol daily corresponding to half or one standard drink a day, respectively.

Model	1929–1949			1949–1970			1970–1990			1990–2005		
	Carbo—hydrates	Fat total	Protein	Carbo-hydrates	Fat total	Protein	Carbo-hydrates	Fat total	Protein	Carbo-hydrates	Fat total	Protein
Corresponding mean	270	78	55	255	84	55	265	62	95	280	58	90
3 nutrients	210	98	70	225	73	110	280	67	70	245	80	75
3 nutrients + alcohol 12,5g/day	-	-	-	220	73	115	330	60	35	230	89	70
3 nutrients + alcohol 25 g/day	-	-	-	225	76	105	285	71	55	285	58	85

### The statistical goodness of the fit of the models

**Fig 6:**
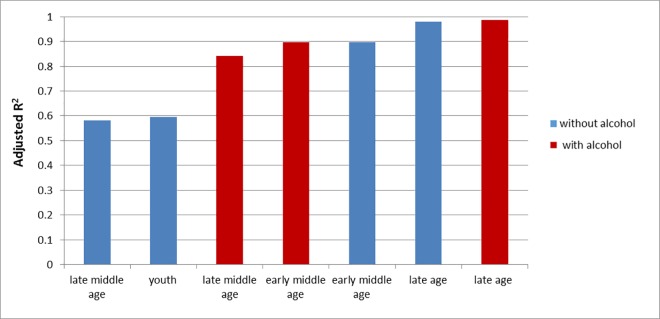
R^2^ adjusted for 7 studied statistical models.

The adjusted R^2^ was used to evaluate the goodness of the fit for the considered models. This parameter protects against overfitting the model and statistical validation with the use of adjusted R^2^ is a standard method for comparing multiple linear regression models that contain different numbers of predictors. Among the considered models we achieved the best statistical fit for “*late age*” period with and without alcohol. The adjusted R^2^ for these models were approximately 0.98. The lowest values of adjusted R^2^ were observed for models: “*late middle age*” and “*youth*”, both without alcohol (see Fig 6). The accuracy of prediction using all models is presented as graphs of predicted R with R original that are included in the S4 File. Fig 6 shows the extent to which our statistical models explain the variability of R_original_. One of the weakest models- youth—explains the variability of R_original_ only in about 60%. It means that diet (macronutrients) accounts only for 60% of the variability of R_original_. Moreover, this model might be also biased by lifestyle factors such as high physical activity that characterized young people and children in the studied period—before the expansion of cars, public transportation, TV and so called “screen time”. Apart from lifestyle changes of the U.S.A. population when we age we have less physical activity and, therefore, the variability of R_original_ may be explained to a larger extent by the diet (adjusted R^2^ at late age above 0.9). On the other hand, the alcohol in the model may to some extent imitate the lack of physical activity and increase the adjusted R^2^. For the youth model about 40% of the variability of R_original_ can be attributed to factors other than diet—most probably physical activity. When we consider early middle age and late middle age models the latter model does not fit this trend, since it explains only slightly less than 60% of the variability of R_original_. We postulate that in the period of 1970–1990 another factor, apart from physical activity, reduced the effect of the diet.

### Alcohol in the diet in different periods of life

Alcohol is accompanying the history of human societies practically from the very beginning. It is well known that when consumed in excess it has detrimental effects on health and often leads to alcoholism. Many sound studies are showing however that in moderate amounts alcohol improves health [13,14]. The Mediterranean diet known for its positive influence on health is characterized by a moderate consumption of alcohol [15,16].

Therefore, to evaluate the influence of alcohol on the optimal proportions of macronutrients we included its consumption in our calculator models

*Youth* model was not considered due to two factors:

Prohibition period in the years 1920–1933

The fact that children are not supposed to consume alcohol. In the original data of alcohol consumption per capita the consumption was calculated for the population aged 15 and older prior to 1970, and for the population aged 14 and older thereafter [10].

In all studied models alcohol in the diet revealed positive influence on R (decreased the absolute value of R). We conclude that in all periods of life except *youth* moderate consumption of alcohol improves health (helps to prevent AD) but the strongest positive influence is observed in the *late middle age* model (see Table 2B, percent of influence).

### Protein in the diet in different periods of life

Protein is required for building and rebuilding our musculature and for the synthesis of a variety of other proteins. Quantitative requirements for protein intake are therefore different in different periods of life—highest in the period of growth and increase of the body mass. Table 3 shows the results of calculations of optimal proportions of macronutrients with respect to reducing the susceptibility of a population to Alzheimer’s disease. Table rows with corresponding mean values show proportions of macronutrients of average availability in a given period, taken with precedence periods. Rows below the corresponding means present our predictions of the best proportions of macronutrients. For *youth* and *early middle age*, in comparison to historical data, we predict beneficial effects of higher consumption of protein whereas for *late middle age* and *late age* we recommend reduction in the percentage of energy coming from protein sources. In models without alcohol, an increase in protein intake in *late middle age* and *old age* exerts a negative influence on R by increasing its absolute value. In models that comprise the consumption of alcohol, protein intake has a similar effect but its negative influence is shifted from *late middle age* to *early middle age* (see Table 2A and 2B) whereas at *late age* an increase in protein intake has a consistently negative influence on R_predicted_.

Interestingly, the “precedence periods” through which protein consumption exerted its biological effects on AD susceptibility equal 5 years for most of the statistical models without alcohol, whereas in models that include alcohol these periods are reduced to 0 years indicating an almost immediate influence of protein intake on the disease outcome when alcohol is consumed on a daily basis.

### Carbohydrates in the diet in different periods of life

Models that do not take into account alcohol consumption, predict that increasing carbohydrate intake exerts positive influence on R (decrease the absolute value of R) for every tested period of life except the period of *youth*. Since the “precedence period” for carbohydrates is 10 years in *youth* this particular model may not be entirely sound because some of the patients have not been born yet. Moreover, this model is one of the weakest statistical significance (see Fig 6).

Alcohol consumption, when included into the models, had no influence on the predicted positive effects of increased carbohydrate intake. In spite of that, all models except the *late middle age* model, predict lowering carbohydrate intake as beneficial for AD prevention (see Table 4, Fig 5).

The precedence periods are 1 year to 15 years and are generally shorter for the later periods of life, probably reflecting the changing biology of the aging organism.

### Fat in the diet in different periods of life

Increase in fat consumption decreases the value of R_predicted_, both in the absence and presence of alcohol, except for the “*early middle age*” model. The precedence period increases with age up to 13 years for the *late age* model. Our predictions point to beneficial effects of the increase in fat consumption in *youth* and especially in *late age* (10% increase in energy share). Addition of alcohol to the statistical models does not change the direction of predictions or their qualitative outcome.

## Discussion

Starting from the correlation between PCPI (1929–2005) and AADR we arrived with the dietary prognosis about proportions of macronutrients that may be optimal for the dietary prophylaxis of AD. The flow chart describing our rationale is presented in Fig 2.

We would like the readership to be aware of all the assumptions we have made to create the basics for the rationale of our model.

The use of death certificates as a measure of AD susceptibility can be criticized due to many confounding factors. However, the oscillations of correlation coefficients R observed earlier [9] are statistically valid and therefore, we proceeded with our analysis described in this paper assuming that, despite all the confounders, we previously observed real changes in the relationship between AADR and income over time so, these changes can be a basis for the present analysis. Detailed discussion of the problem of confounding factors is contained in our previous paper and we refer interested readers to the appropriate fragments in Stępkowski et al. 2015 [9].

The main assumption made, that availability of macronutrients is proportional to consumption, justified the use of data for the whole population of the U.S.A. for which such data are available. Moreover, as we obtained reasonable results concerning proportions of the macronutrients in the diet, we believe that indeed average macronutrient consumption is proportional to its availability.

Another assumption that was particularly crucial to obtaining the “calculator equations” is the postulation that the variability of R_original_ can be explained by the availability of macronutrients taken with precedence periods. It is possible that any parameter whose variability would resemble the variability of R could be used to fit the equations, however, we propose that diet (macronutrients proportions) is the most probable biological cause of the previously observed variability in the strength of correlation between PCPI and AADR. Studies on animals and humans support the view that proper balance of macronutrients is important for maintaining health [2,3,6]]. Using optimized precedence periods we obtained very good statistical parameters of fit. This is not surprising since the optimization of the precedence periods relied on finding the best fit for different precedence periods. However, we think that this is the only possible way to determine the real precedence period for biological effects of a particular macronutrient based on its historical availability.

Determination of the macronutrient proportions is based on the smallest difference between the total energy of prognosis and the total energy of macronutrients (if) consumed according to their availability in a given period of time with regard to the precedence periods. This seems to be the optimal strategy to obtain predictions which are closest to the historical consumption but also postulate changes in the diet significant enough to reduce the risk of Alzheimer’s disease. Our predictions are in line with US guidelines for food consumption that have been recently published [17]. In particular, our data support the conclusion that higher average intake of fat and lower of protein in the last part of life brings health benefits.

### The predicted AD preventive changes in the proportions of macronutrient intake at a particular age versus historical macronutrient intake

#### Youth

The model predicts reduction in carbohydrates consumption (12% in energy share), increase in fat consumption by 9% and 3% increase in protein consumption.

#### Early middle age

The model predicts reduction in carbohydrates by 6%, reduction in fat consumption by 5% and significant increase in protein consumption by 11%

#### Late middle age

The model predicts moderate reduction in protein consumption by 5%

#### Late age

The model predicts significant reduction in carbohydrates by 7% and increase in fat consumption by 10% and small reduction in protein intake.

Concluding, the predicted prophylactic solution i.e., the increase in fat intake in *youth* may represent the need for higher energy expenditure accompanied by high brain requirement for lipids. On the other hand, in *late age*, higher fat intake may be beneficial to meet the decreasing metabolic efficiency of the aging brain [18]. We have obtained results showing the necessity to reduce total fat consumption in early midlife and late midlife whereas in youth and late life we propose to increase fat consumption relatively to other macronutrients. To understand better these predictions let us consider the influence of the presence of fat in the diet on the brain. Appearance of fat in the diet during the evolution affected the development of the brain of the hominids [19]. The dry mass of the brain consists mostly of lipids [20] so it is only natural that it requires a large supply of lipids to function and thrive. During development of the organism the kind and the amount of fatty acids in the diet affects the lipid composition of the brain [21]. Moreover, the lipid composition of the brain in childhood is most probably significant for the late life susceptibility to neurodegenerative diseases [21]. Of note, the lipid composition of the cell membrane influences the activity of gamma secretase [22] and, as a consequence, may impact Aβ production.

Therefore, in the old age, when the brain function begins to deteriorate, the presence of fat in the diet is probably important for preserving the cognitive function of the brain. In the context of increased longevity and susceptibility to AD and other diseases related to age it becomes all the more relevant what kind of fatty acids are delivered with food [7,8,23].

As it has been mentioned above, our results indicate increased demand for fatty acids in the first quarter of life, which is quite obvious, and in the last quarter of life which is less obvious. We interpret this as follows: in the late life the energetics of the brain is disturbed by impaired glucose metabolism ([18] and articles reviewed by Cunnane et al.[24]). In such a situation, by eating larger amounts of medium chain fatty acids, it is possible to support the energetics of the brain with ketone bodies instead of glucose [1]. Medium chain fatty acids are known to shift the brain into a moderate state of ketosis. Thus, both the composition of lipids and the amount of fat in the diet seem to have influence on brain performance in late age. Janssen et al [25] have reported that aged female mice, on “high” fat diet (19% butter), performed better than aged female mice on “normal” diet (3,3% fat), in spatial learning tests. This observation is in line with our findings. Only few epidemiological studies link total fat consumption with susceptibility to dementia and AD [6,26]. Luchsinger et al. found negative influence of fat only for apolipoprotein E epsilon4 allele carriers [27]. Data reported in reference [27] concern food intake at one time point only. Since food intake patterns do not change so dramatically we can assume that they were similar in midlife and in older age unless a change has been imposed by a serious illness. Thus, results of those studies depends also on the food intake in previous years (i.e. also on midlife intake). Our results point to reduction of fat intake in midlife and are in agreement with studies of Luchsinger et al. [27], Laitinen et al. [26] and with studies on rodents reported by Kadish et al [28] and Raider et al.[29]. We propose only a modest shift in the consumption of fat, from the historical one, which we believe can reduce the AD susceptibility of a population, including also apolipoprotein E epsilon 4 allele carriers.

In this paper we relate the consumption of total fat, along with other macronutrients, with the susceptibility of the U.S. population to AD. Due to limitations of our statistical approach it was not possible to study prophylactic effects of particular types of fat. Nonetheless, several studies have shown a positive effect of total fat consumption in old age both with regard to all-cause mortality [7,8] and MCI prevention [6]. On the other hand, specific components of total fat have definitely different effects on the susceptibility of a population to AD ([23]and references therein) and all-cause mortality [7,8], but due to synergistic or negative effects of other diet components their influence should be addressed in the context of the diet as a whole [23].

Our results predict a preventive influence of light to moderate alcohol consumption on the susceptibility of a population for AD. This is generally in line with the current knowledge on the effects of such drinking pattern for prevention of dementia including AD [30]. We predict such influence for an average statistical American. Some differences may appear for ApoE ε4 allele carriers and dependence on the type of alcohol may also exist resulting, however, in”no effect” rather than “negative effect” [30]. Since, we have used the total intake of ethanol for regression analysis (best scoring by comparison with types of alcoholic beverages–data not shown) our results do not account for the effect of the types of alcoholic beverages. It should be also noted that the calculated “standard drink” corresponded to 25 g of ethanol and differed from the standard American drink which is 14 g of ethanol [31]. This, however, does not influence our qualitative conclusion about preventive effect of moderate alcohol intake on the susceptibility of the American population to AD.

Our predictions are based on the assumption that diet can influence pathological processes occurring in the brain. Such influence in humans is well documented in the case of the so called ketogenic diet (low carbohydrates and high fat) effective in some cases of epilepsy [32] and supposedly promising in the treatment of AD [1,33]. The recently published dietary guidelines for US allow for higher fat intake [17,34]. Our predictions are in line with those guidelines as we propose a prophylactic solution of higher fat intake in *youth* and *late age*.

*Youth* and *early middle age* are characterized by another predicted prophylactic solution—the higher protein intake. Nevertheless, later in life, the needs for protein are smaller, exactly as our models predicts.

Generally, all models apart from *late middle age* predict reduction in carbohydrate intake. Since, we calculated our models from AD death rates, and AD shares some pathological features with T2D [35] we think that our results are also in line with the guidelines for prevention of T2D, especially with regard to lower intake of carbohydrates [36]. On the other hand we observed that, with the exception of youth, carbohydrates have positive influence on R (lowering absolute R value), which does not corroborate our finding that optimal diet should have a reduced amount of carbohydrates. This discrepancy is probably caused by the fact that our algorithm, based on minimum energy difference of prediction from the energy corresponding to mean (for each period) historical availability of macronutrients, calculates an equilibrium between three macronutrients and the optimal equilibrium is forcing decrease in carbohydrates consumption.

### The precision of predictions

One may ask whether the proposed shifts in the macronutrient proportions will be effective in reality? First of all, they are in line with the animal studies [2,3] predicting high protein consumption at the reproductive age and low protein consumption for achieving longevity. Our predictions can be partially tested on the results of human studies published by Roberts et al.[6]. The authors calculated hazard ratios (HR) for mild cognitive impairment (MCI) of different proportions of macronutrients in a diet of aged population. They found that for aged people, corresponding roughly to our late age period, lower intake of carbohydrates, higher intake of total fat and higher intake of protein was beneficial in terms of developing MCI. Our results agree well with their data. In particular, our predictions for late age without alcohol consumption correspond to the Q2 quartile of carbohydrate consumption (beginning of the increasing trend of higher risk for MCI) in Roberts et al.[6], with HR of 0.99 (CI 0.64–1.55) or 0.91 (CI 0.55–1.50, for fully adjusted model. For total fat our predictions correspond to the Q4 quartile with HR of 0.58 (CI 0.39–0.88) or 0.56; (CI 0.34–0.91). We predict reducing the consumption of protein to 15%, which corresponds to the referent quartile Q1 i.e. beginning of an increasing trend of MCI risk reduction in Roberts et al. The apparent inconsistency may stem from the fact that our studies refer to AD not MCI or, alternatively, that 15% content of protein in the diet is just 1% less than in the Q2 quartile in Roberts et al., in which protein provides 16–18% of energy supply and which represents a reduced risk of MCI, with HR of 0.70 (CI 0.47–1.03) or 0.60 (CI 0.39–0.91 for fully adjusted model). 1% difference in energy units may be an acceptable bias of our method. Taking the hazard ratios from Roberts et al.[6] (corresponding to our predictions) we can calculate an average HR (from fully adjusted models) as 0.69; thus, the proportions of macronutrients that we have predicted may reduce the risk of AD. This conclusion is corroborated also by the fact that, in the case of fat and protein, the predicted proportions correspond to the quartiles in Roberts et al. [6] with HR values pointing to a statistically significant reduction of risk of MCI and, in the case of carbohydrates, to the quartile with the lowest risk (the beginning of a statistically significant trend for increasing risk). In summary, based on the comparison of our analysis with the results of solid epidemiological data of Roberts et al.[6] we conclude that our predictions concerning late age diet are highly precise.

However, to confirm the effectiveness of the proposed changes in a diet in reducing the risk of AD a long term longitudinal studies should be performed on a population of healthy subjects. Our predicted proportions of macronutrients possibly lowering the chance of AD development, may be also used to divide the subjects of the previously published studies (for example described in [15,37,38]) to new trial groups according to macronutrient consumption and to reanalyze the statistical data to give new insights into the effectiveness of those particular diet changes in prevention of AD.

### Strengths and Limitations

Our approach to the problem of AD prevention is based on whole population data. Traditional epidemiological approach is, on the contrary, based on studies carried on selected subpopulations and therefore is subjected to errors caused by extrapolation of the data to the whole population and by the selection criteria and using the food questionnaires. On the other hand whole population data do not take into consideration geographical differences, migrations, use mean per capita data etc. All the confounding factors mentioned in our previous paper [6 and references therein] tend to reciprocally reduce their influence and we observe reasonable prediction as to the ratio and amounts of macronutrients. Our predictions are rather evolutionary than revolutionary and therefore seem to be safe to introduce to the society nutritional behaviors [28].

## Supporting Information

S1 File Tables presenting predicted R values, total energy of macronutrients in a given proportion, and the proportion of macronutrients with minimum total energy difference from the average total energy of macronutrients in a given period. (XLSX)

S2 File Active spreadsheet with the calculator. (XLS)

S3 File Manual for the tables of macronutrient proportions and the calculator spreadsheet. (DOC)

S4 File Graphs illustrating the accuracy of prediction with predicted R and original R. (DOC)
