# Correction: The Calculator of Anti-Alzheimer's Diet. Macronutrients

**DOI:** 10.1371/journal.pone.0209723

**Published:** 2018-12-19

**Authors:** Marcin Studnicki, Grażyna Woźniak, Dariusz Stępkowski

Following publication of our work [1], concerns were raised about some detailed aspects of the calculator statistical model. The critiques were related both to the rationale and the implementation of the model.

In response, we have introduced global optimization of precedence periods instead of a sequential one and recalculated the results using updated table of nutrients (USDA) as well as corrected set of “R_original_” [2]. In this Correction, we explain these updates and provide revised Tables and Figures to correct the errors identified in the published article.

1. One of the purposes of this correction was to recalculate the results using the updated table of nutrients availability (Source: Calculated by USDA/Center for Nutrition Policy and Promotion. Data last updated Feb. 1, 2015). The Table was accessed for the purpose of recalculation in March 2017 and during the process of preparing the text of Correction it was replaced by USDA CNPP for a new one (https://www.cnpp.usda.gov/USFoodSupply-1909-2010) which does not differ in respect to macronutrients availability. Please see attached the set of nutrients data used by the authors (S1 File). In the previous version of the paper [1] the outdated table was used which is currently not available. The updated table of nutrients differs from the previous version and impact on the recalculated results.

2. Concerns were also raised about the previously established set of the values of “R_original_” [2]. Those values were checked and minor errors were found. These errors are listed below and did not change the results and conclusions of the paper [2]:

Years published corrected

1952    –0.567    –0.526

1959    –0.582    –0.583

1960    –0.567    –0.587

1982    –0.503    –0.501

1983    –0.534    –0.535

1984    –0.550    –0.551

2004    –0.488    –0.487

These errors occurred due to the manual entry of data, as it was difficult to judge to which extent the errors influenced the results described in the paper [1]. Accordingly, we recalculated the entire dataset using the corrected set of “R_original_”. The recalculation impacted Fig 1 and Tables 1–4. Please see the corrected Fig 1 and Tables 1–4 and captions here.

**Fig 1:**
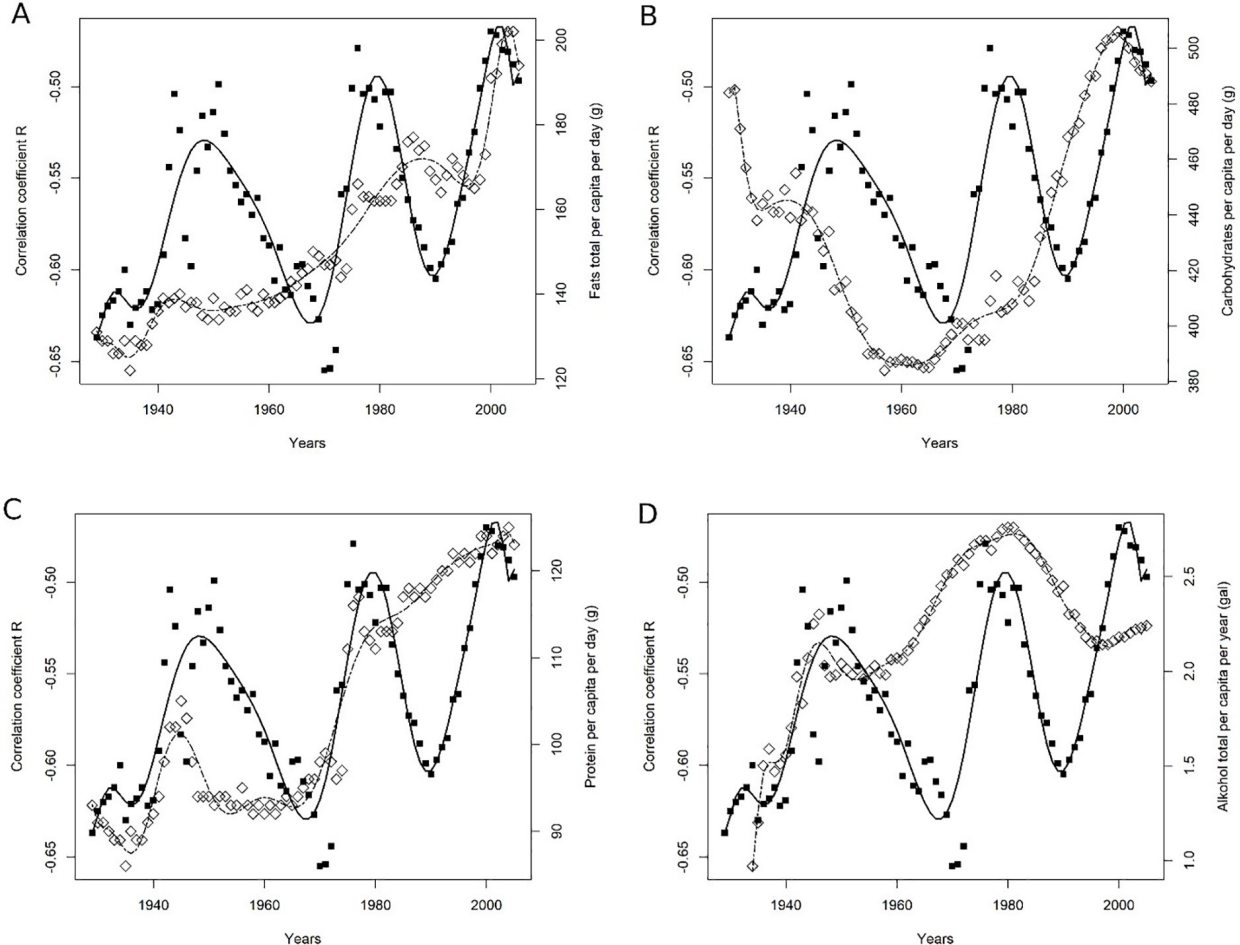
**ABCD** The time course of the availability of four macronutrients and variability of R in the period 1929–2005. A Fat total, B Carbohydrates, C Protein, D Alcohol total.

**Table 1:** Regression coefficients for three macronutrients without alcohol (A) and three macronutrients with alcohol (B) and their corresponding periods of precedence in years.

A												
Variable	1929–1949			1949–1970			1970–1990			1990–2005		
	Period of precedence	b	p value	Period of precedence	b	p value	Period of precedence	b	p value	Period of precedence	b	p value
Intercept		-0.5570			-0.7104			3.0645			-2.1715	
Carbohydrates	10	-0.0009	0.04604	6	0.0017	<0.00001	19	-0.0069	<0.00001	2	0.0015	<0.00001
Fat	19	0.0068	0.00018	10	0.0019	0.07448	11	-0.0152	<0.00001	15	0.0018	0.00018
Protein	1	-0.0046	0.02127	0	-0.0088	<0.00001	16	-0.0152	<0.00001	12	0.0052	0.00003
B												
Variable	1929–1949			1949–1970			1970–1990			1990–2005		
	Period of precedence	b	p value	Period of precedence	b	p value	Period of precedence	b	p value	Period of precedence	b	p value
Intercept					-0.2859			3.4672			-1.0961	
Carbohydrates				6	0.0016	<0.00001	19	-0.0088	<0.00001	1	0.0008	0.00005
Fat				5	-0.0010	0.24823	8	-0.0032	<0.00001	15	0.0012	0.00009
Protein				0	-0.0103	<0.00001	0	0.0045	<0.00001	13	0.0028	0.00005
Alcohol				6	0.0735	0.00075	2	-0.2247	0.01220	5	-0.1526	<0.00001

**Table 2:** The values (positive or negative) of macronutrient coefficients and the strength of the influence (in %) of each macronutrient in each model on R_predicted_. **The strength of influence was calculated using so called “standardized correlation coefficients”.** Bold font indicates the nutrient with the highest influence on R_predicted_. (A) models without alcohol. (B) Models with alcohol.

A								
Period	Carbohydrates		Fat		Protein			
	Influence	%	Influence	%	Influence	%		
1929–1949	Negative	21.42	Positive	**49.60**	Negative	28.98		
1949–1970	Positive	**67.13**	Positive	7.94	Negative	24.93		
1970–1990	Negative	**41.61**	Positive	28.39	Negative	30.00		
1990–2005	Positive	**37.07**	Positive	28.70	Positive	34.23		
B								
Period	Carbohydrates		Fat		Protein		Alcohol	
	Influence	%	Influence	%	Influence	%	Influence	%
1929–1949								
1949–1970	Positive	**56.56**	Negative	4.13	Negative	25.94	Positive	13.37
1970–1990	Negative	**40.54**	Negative	20.09	Positive	24.74	Negative	14.63
1990–2005	Positive	23.56	Positive	12.53	Positive	14.90	Negative	**49.01**

**Table 3:** Parameters of goodness of fit for models without alcohol (A) and with alcohol (B).

A				
Goodness of fit statistics	Period			
	1929–1949	1949–1970	1970–1990	1990–2005
Correlation coefficients R	0.8969	0.9730	0.9736	0.9953
Coefficient of determination R2	0.8044	0.9468	0.9479	0.9907
Adjusted *R*^2^	0.7699	0.9379	0.9387	0.9883
F test	23.3	106.7	103.1	424.7
p-value	0.0001	0.0001	0.0001	0.0001
Standard error of prediction	0.0203	0.0098	0.0133	0.0053
B				
Goodness of fit statistics	Period			
	1929–1949	1949–1970	1970–1990	1990–2005
Correlation coefficients R		0.9844	0.9783	0.9988
Coefficient of determination R2		0.969	0.9571	0.9976
Adjusted *R*^2^		0.9617	0.9464	0.9968
F test		132.7	89.21	1164
p-value		0.0001	0.0001	0.0001
Standard error of prediction		0.0077	0.0124	0.0028

**Table 4:** Comparison of the mean availability of macronutrients in the corresponding periods of precedence with predicted optimal proportions of macronutrients in grams per day assuming 2000 kcal diet. Calculated from models without alcohol and with two levels of alcohol consumption: 12.5 or 25 g pure ethanol daily corresponding to half or one standard drink a day, respectively. It should be mentioned that standard drink that we have used differs from US standard drink which contains 14g of pure alcohol.

Model	1929–1949			1949–1970			1970–1990			1990–2005		
	Carbo—hydrates	Fat total	Protein	Carbo-hydrates	Fat total	Protein	Carbo-hydrates	Fat total	Protein	Carbo-hydrates	Fat total	Protein
Corresponding mean	266	79	56	245	87	59	234	91	62	242	88	60
3 nutrients	258	95	29	267	93	24	248	97	33	226	82	90
3 nutrients + alcohol 12,5g/day	-	-	-	265	92	27	216	96	68	221	80	99
3 nutrients + alcohol 25 g/day	-	-	-	262	91	33	196	91	98	218	77	109

3. Use of sequential versus global optimization was questioned. Contrary to our assumptions, sequential optimization procedure does not cover all possibilities of a highest maximum of correlation—our criterion for choosing appropriate precedence periods. Therefore, we re-applied the procedure of determining precedence periods (using global optimization) up to -20 years except the period 2 (1949–1970) with the presence of alcohol for which we applied shorter precedence periods (-15 years) due to years of prohibition in the USA.

Results of recalculation that incorporate the above changes are presented in the new versions of Fig 1, Fig 5, and Tables 1–4. Recalculation results differ from the original version of the paper [1]. The most pronounced differences concerned prediction of the proportions of macronutrients in Table 4 and Fig 5. Please see the corrected Fig 5 and caption below.

**Fig 5:**
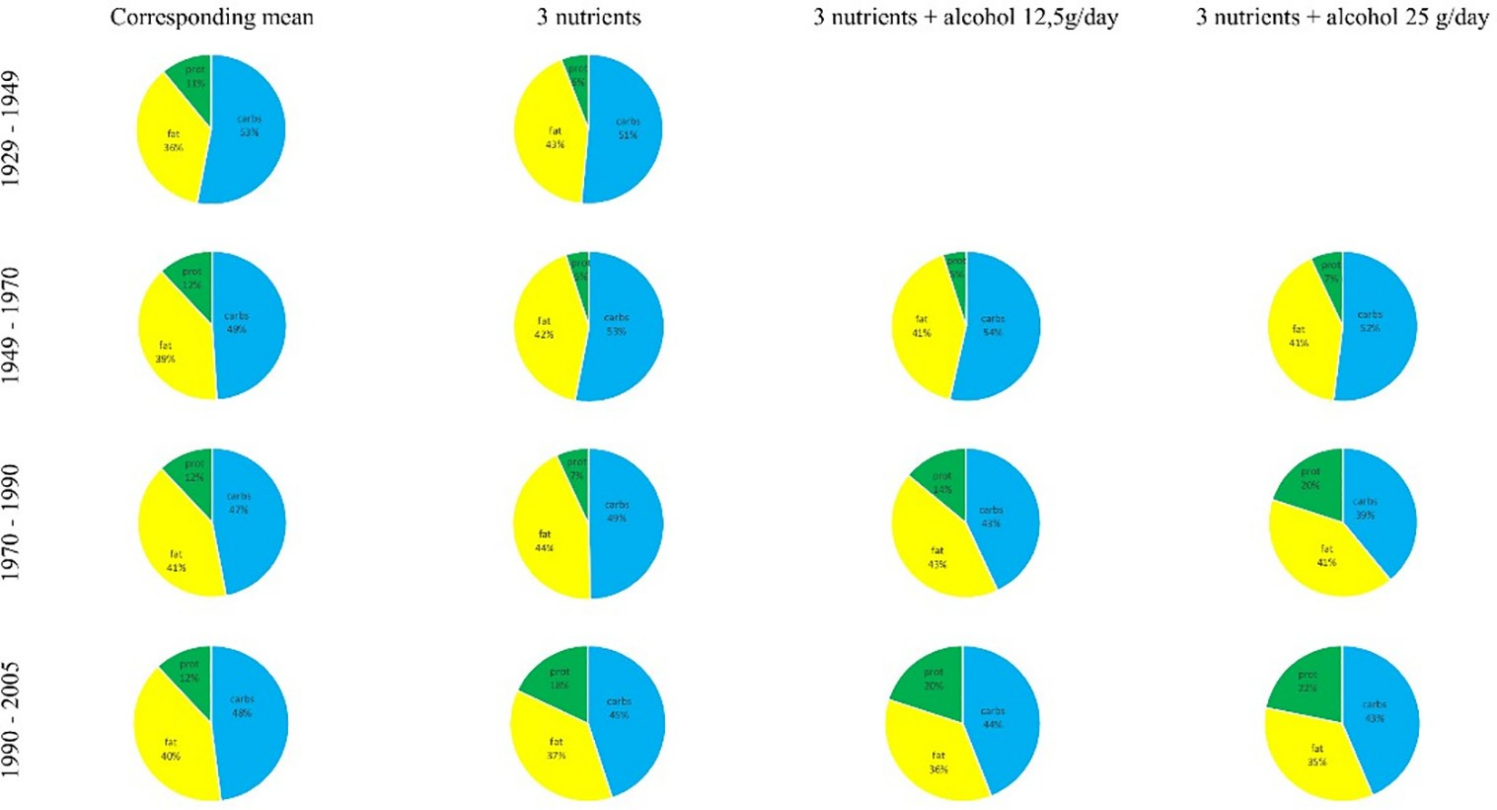
Wheel charts of the recalculated proportions of macronutrients in percent of the energy units for each period studied.

Revised results concerning protein share in the diet differ from those presented in the previous version of the paper [1]. We currently postulate to reduce the amount of protein in the diet for the first three quarters of life. Contrary, for the late age we propose to consume more protein than in the first three quarters of life and more than the historical availability for the studied population (Table 4 and Fig 5).

Including alcohol into calculations caused an increase in the predicted protein consumption for the period of late middle age. When it comes to carbohydrates and total fat consumption our predicted intakes are moderately lower than the corresponding mean for carbohydrates and on the same level or slightly higher as mean for total fat. It should be mentioned that total fat is a nutrient with highest influence on R_predicted_ for most of the models (see Table 2). Since we applied 5g step size in the calculator the ±5g is a maximal accuracy of our predictions.

## Methods: Additional explanations

The formula for calculating the energy difference for finding the minimum difference:

Since our “calculator” produces many results with R_predicted_ within the range (0, -0.1) yellow coded, we imposed on it criterion of minimal energy difference of a given hand-made triple set of nutrients amounts from mean availability taken with appropriate precedence periods. The energy difference is calculated using the following formula:

Edifference = (Abs((mean_nut_1 * cal_nut_1)—(cal_nut_1 * nut_1(i))) + Abs((mean_nut_2 * cal_nut_2)—(cal_nut_2 * nut_2(j))) + Abs((mean_nut_3 * cal_nut_3)—(cal_nut_3 * nut_3(k))) + Abs((mean_nut_4 * cal_nut_4)—(cal_nut_4 * nut_4))) * 0.7) where Abs means absolute value, cal_nut_x means caloric value for nutrient x. Factor 0.7 is introduced to make a shift from availability to more real consumption assuming 30% losses.

We have also fixed an error which occurred in our results presented in Table 4 caused by replacing data columns for fat with that for protein and vice versa for periods 1970–1990 and 1990–2005.

In addition, the authors provide the following clarifications:

1. In regard to the concern of the reader that the calculator produces an infinite number of solutions—we agree, that in theory, there is an infinite number of solutions but due to the discrete step sizes of our calculator this number is limited. Imposing the criterion of minimum energy difference reduces the number of possible solutions to a very few and with larger step sizes (like 5g which seem reasonable taking into consideration the precision of input nutrient data) to only one. The criterion is based on the rule of keeping the predictions as close as possible to the set of nutrients on which the regressions were done. Our criterion of the minimum energy difference between given pattern and mean availabilities enables that. Therefore, such a selection of one solution has the highest confidence from all possible.
2. The scaling using energy units has been done to express proportions of macronutrients in a more applicable form than availabilities (Table 4 and Fig 5). Such an approach is often used in the nutritional sciences. To compare macronutrients intake the equivalent energy is calculated. There is a quite strong biochemical background supporting the consideration of proportions rather than amounts of macronutrients [3]. Many thanks to the reader for pointing out that scaling can change the (R_predicted_). Although we have not explained it in detail it was clear for us, but we thought that it was not important, since the calculator works on availabilities just to find optimal proportions of macronutrients and these proportions can be transferred to 2000 kcal diet. Application of the equation of calculator to a different set of scaled amounts is not allowed. So, we think that our 2000 kcal diet is valid.
3. The ranges of validity of the model are an important issue. According to our assumptions valid range is that which give reasonable R_predicted_ i.e. (within range -1, 0). One can imagine a diet which taking into account nutrient availabilities, gives values outside of this range. It could be interpreted as a very unhealthy. This does not concern scaling with the same proportions. Diets outside the range (-1, 0) have proportions located far away from the optimal.

## Discussion

The main assumption we have made, that the remarkable oscillations of the R_original_ observed in the paper [2] could be explained by the variations in the proportions of macronutrient consumption by the population of the USA, need to be confirmed by standard epidemiological studies. Recently, several papers appeared which suggest that our predictions concerning protein consumption in different periods of life and its relation to cognition decay in old age [3–5] are valid.

## Supporting information

S1 File Nutrients data. (XLSX)
