# Supplementary material for: The Calculator of Anti-Alzheimer’s Diet. Macronutrients
Source: PLoS One. 2016 Dec 19;11(12):e0168385. doi: 10.1371/journal.pone.0168385 (PMC5167378; doi:10.1371/journal.pone.0168385)

User Manual for the anti-Alzheimer’s Diet Calculator

Step 1. Insert the parameters from linear regression (from appropriate model) into the column marked “Model”. The diet calculator is designed for a maximum of 5 nutrients total. In case of a smaller number of nutrients, please leave the remaining cells blank.

Step 2. Insert the energy values for nutrients in kcal per gram in the Kcalc column.

Step 3. Enter the information about the start value of daily nutrient consumption, the magnitude of the step by which the consumption will be changed and number of step for each individual nutrient. Additionally, insert the historical mean of daily consumption corresponding to the appropriate model. It will be used to obtain the minimum energy difference between the historical mean daily consumption (availability) and the calculated diet.

Step 4. For nutrients 4 and 5 you can calculate the diet assuming constant daily consumption of these nutrients

Step 5. In the selected area enter the minimum value of the observed R (lower R) and the maximum of the observed R (upper R).

Step 6. To start calculation of the diet click on the blue box.

Results

The rows represent a range of Nutrient 1 values and columns represent the range of Nutrient 2 values. Subsequent tables correspond to the change in the value of Nutrient 3. The results calculated for one combination of values of daily consumption of Nutrient 1 and Nutrient 2 are shown in two columns. The first column presents the obtained values of R. The second column shows values of the total energy (in kcal) of the considered combination (proportions) of nutrients.


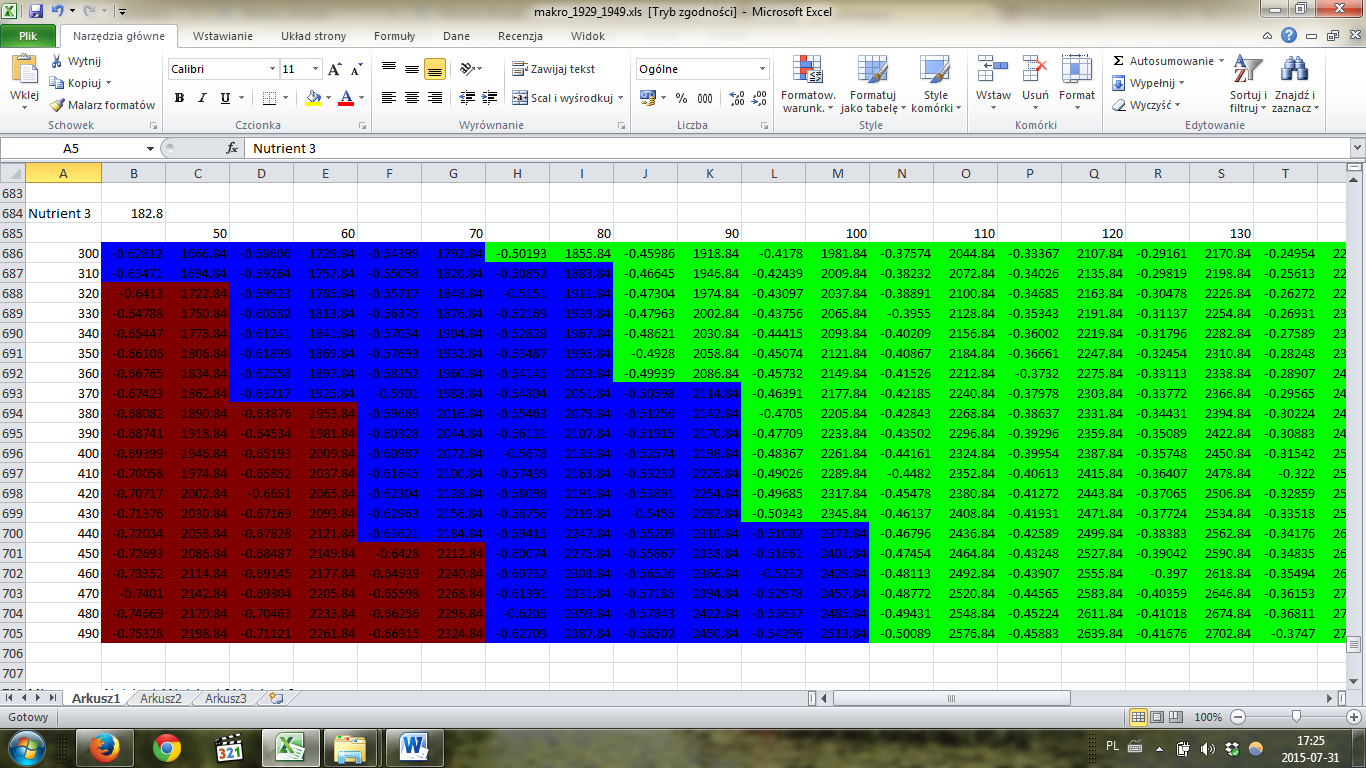


Explanation of the color code is shown in the table below:

| Color cell | Value of R |
| --- | --- |
|  | Absolute calculated value of R above the observed absolute value of R. |
|  | Absolute calculated value of R in the range of the observed absolute value of R. |
|  | Absolute calculated value of R lower than the observed absolute value of R in the range |Robs| - |-0.1|. |
|  | Optimal range of R between -0.1 and 0 |
|  | Calculated R above 0 |

At the bottom of the table the minimum difference between the total energy of the calculated diet and the energy of the historical diet (in kcal) is shown. The combination of nutrients with minimum value of energy difference from all tables indicate the optimal composition of a diet corresponding to a given period of life (based on particular regression model).


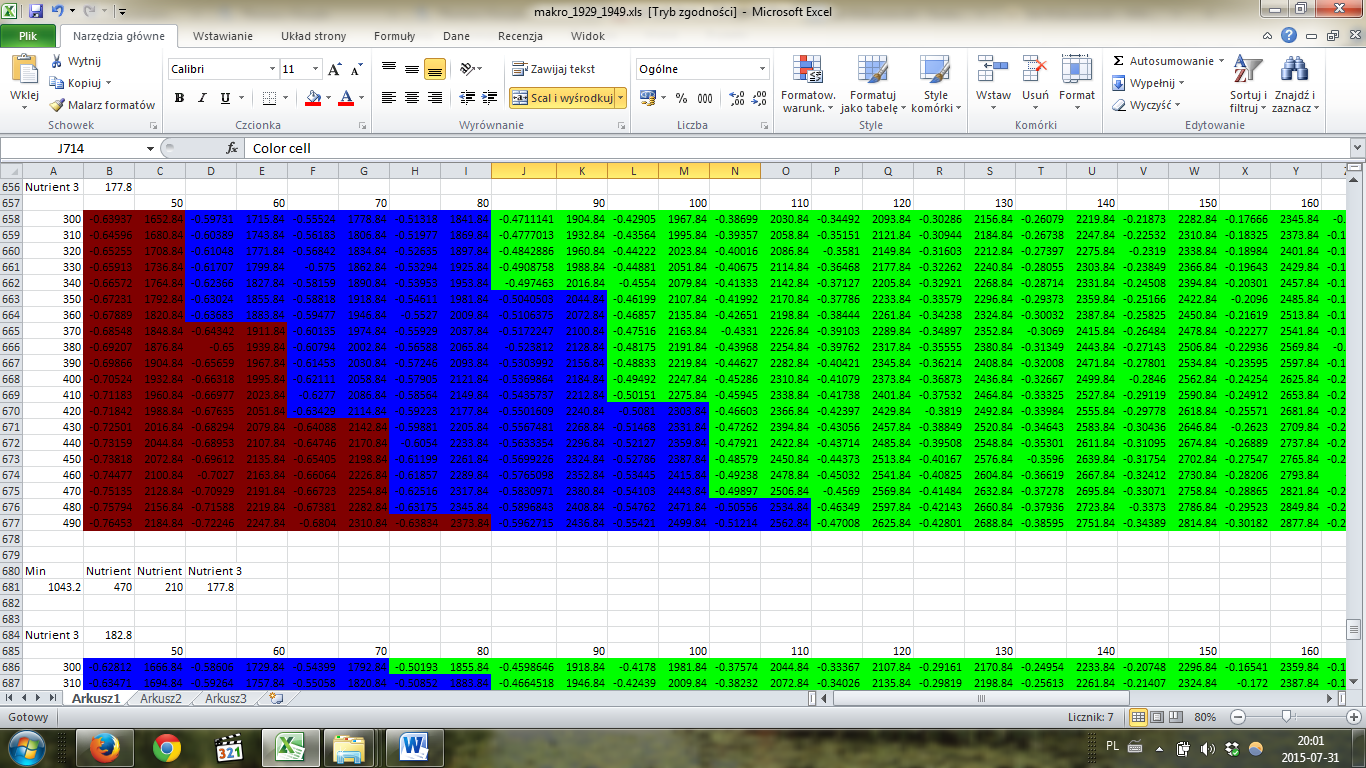

Supplement: S3 File — (DOC) [file pone.0168385.s003.doc]
