# Supplementary material for: The Calculator of Anti-Alzheimer’s Diet. Macronutrients
Source: PLoS One. 2016 Dec 19;11(12):e0168385. doi: 10.1371/journal.pone.0168385 (PMC5167378; doi:10.1371/journal.pone.0168385)
Supplement: S4 File — (DOC) [file pone.0168385.s004.doc]

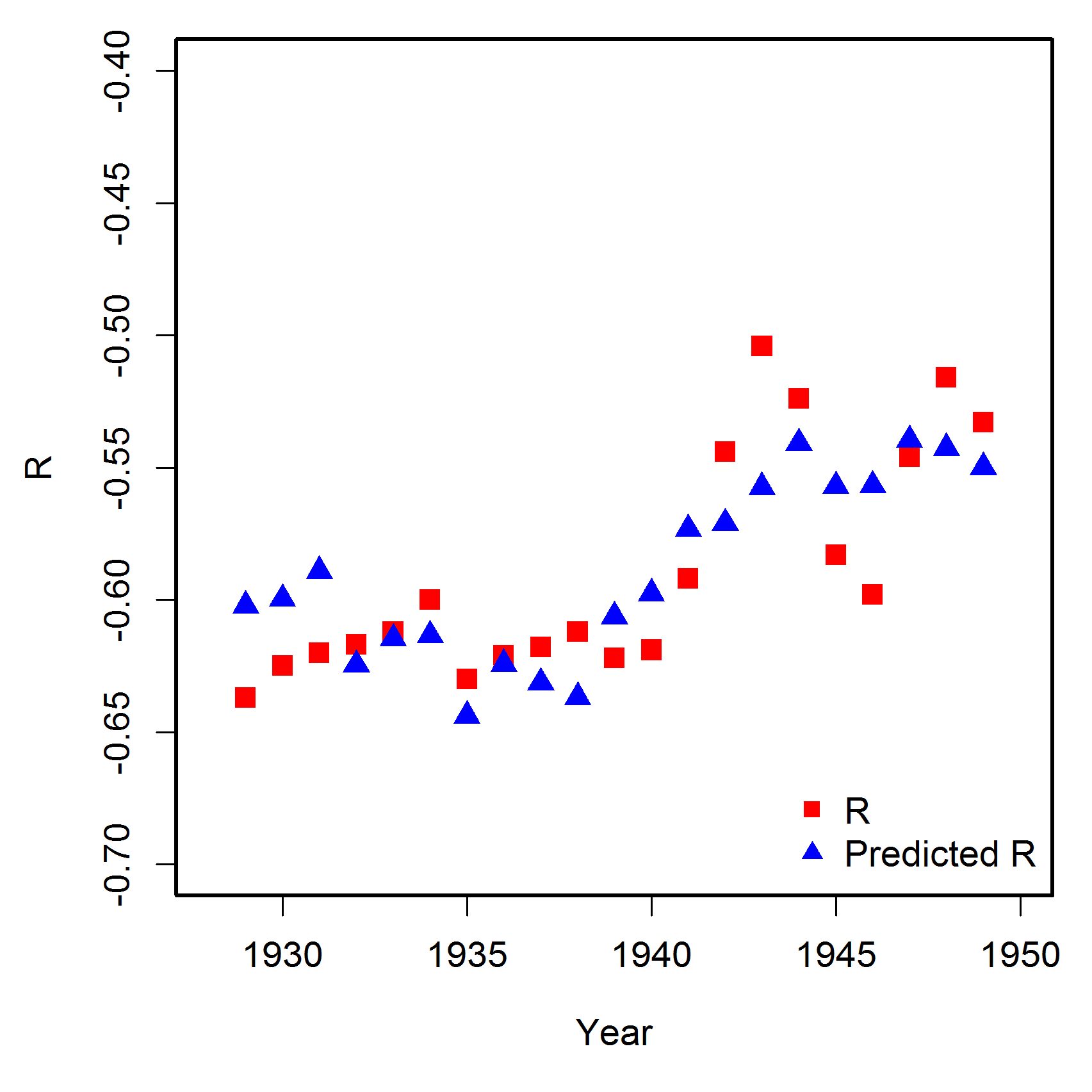


Figure 1. Assessment of the goodness of model fit for the 1929-1949 period without alcohol consumption


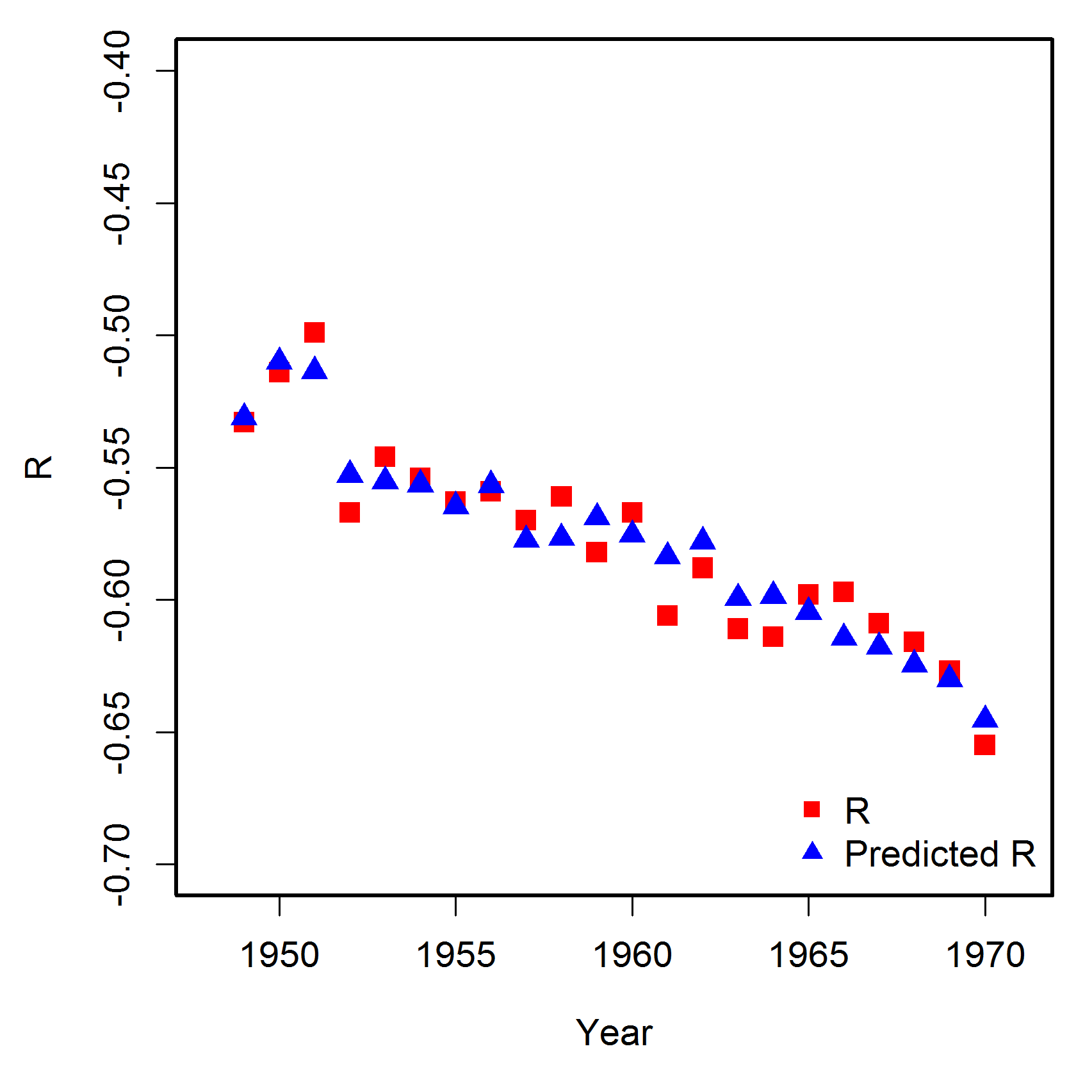


Figure 2. Assessment of the goodness of model fit for the 1949-1970 period without alcohol consumption


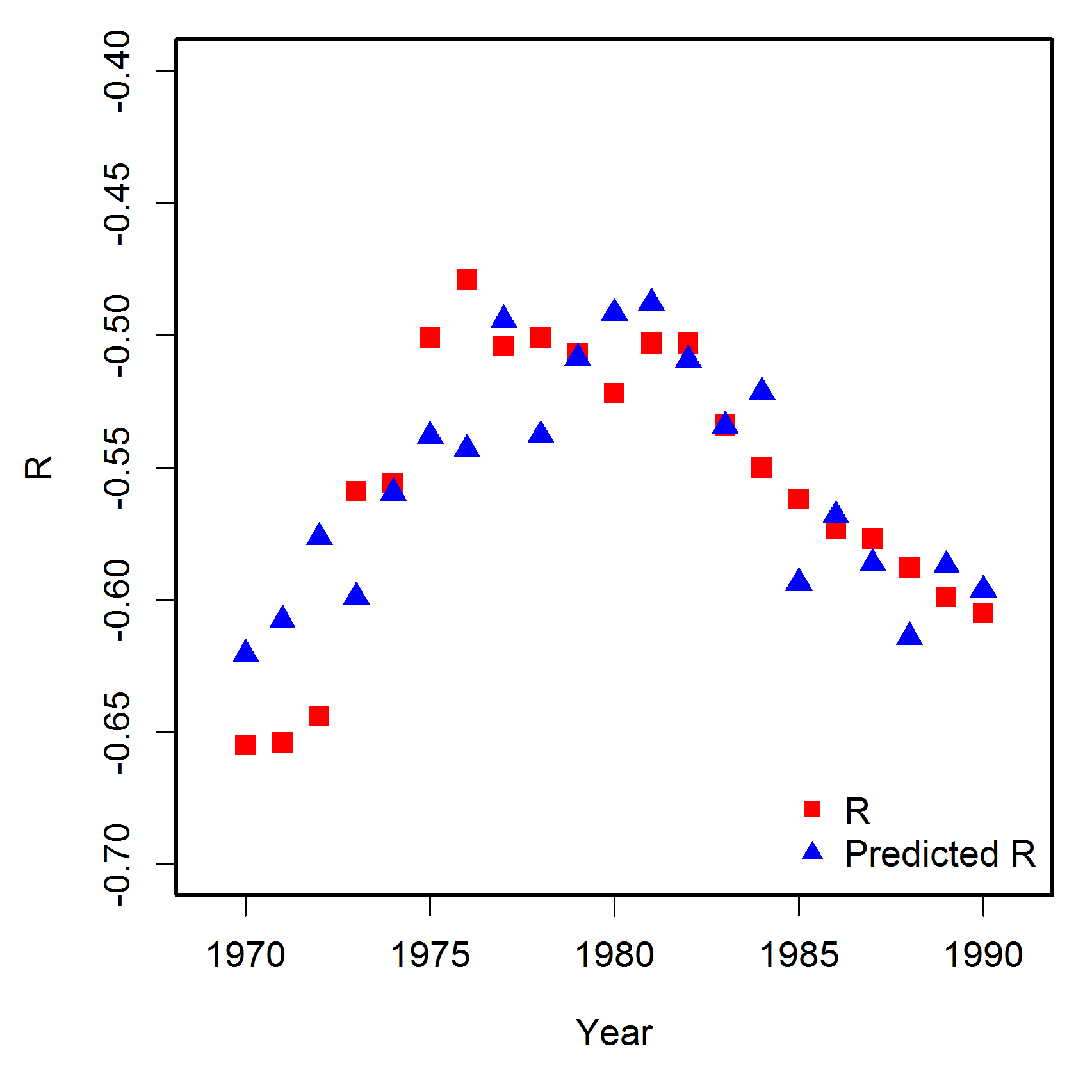


Figure 3. Assessment of the goodness of model fit for the 1970-1990 period without alcohol consumption


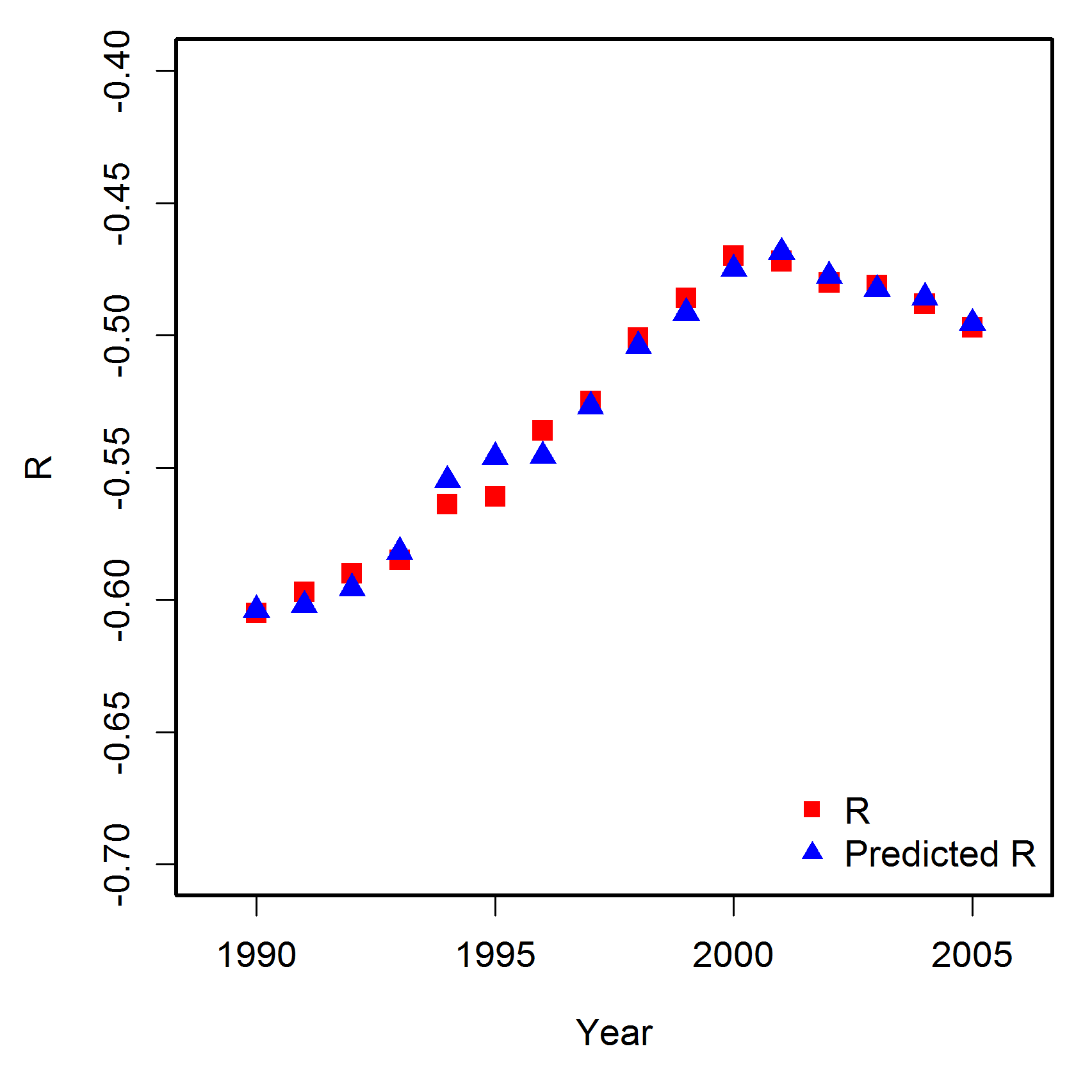


Figure 4. Assessment of the goodness of model fit for the 1990-2005 period without alcohol consumption


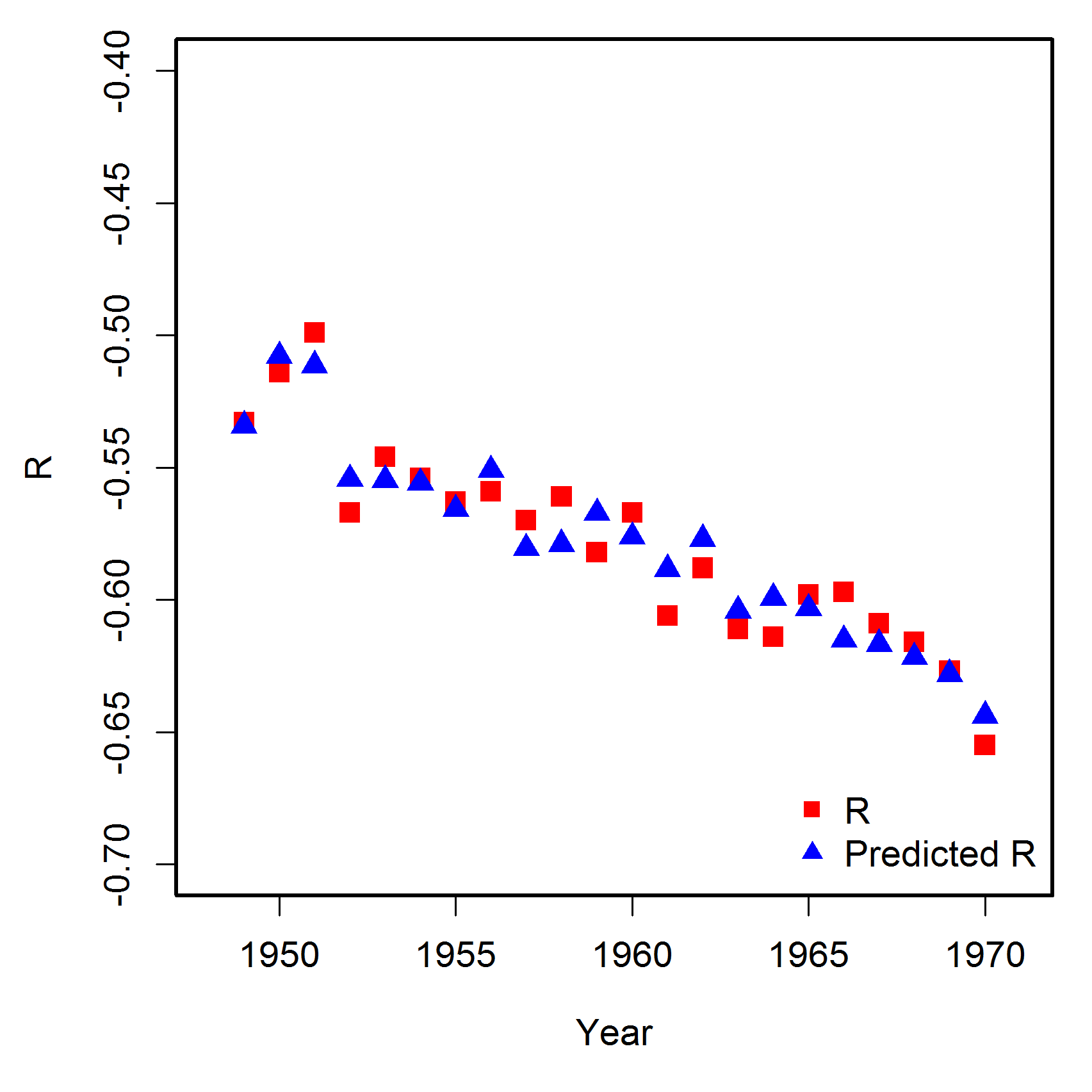


Figure 5. Assessment of the goodness of model fit for the 1949-1970 period with alcohol consumption


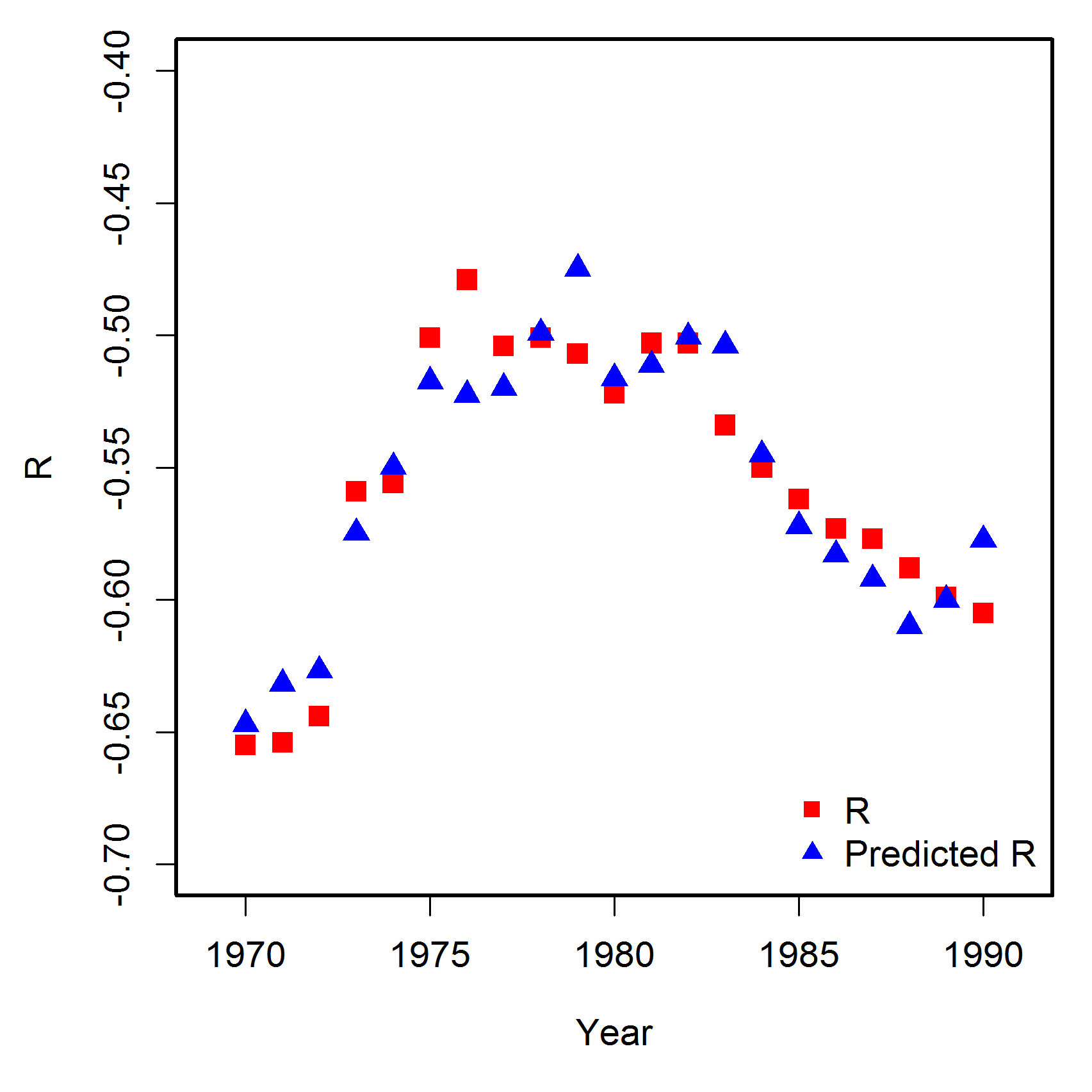


Figure 6. Assessment of the goodness of model fit for the 1970-1990 period with alcohol consumption


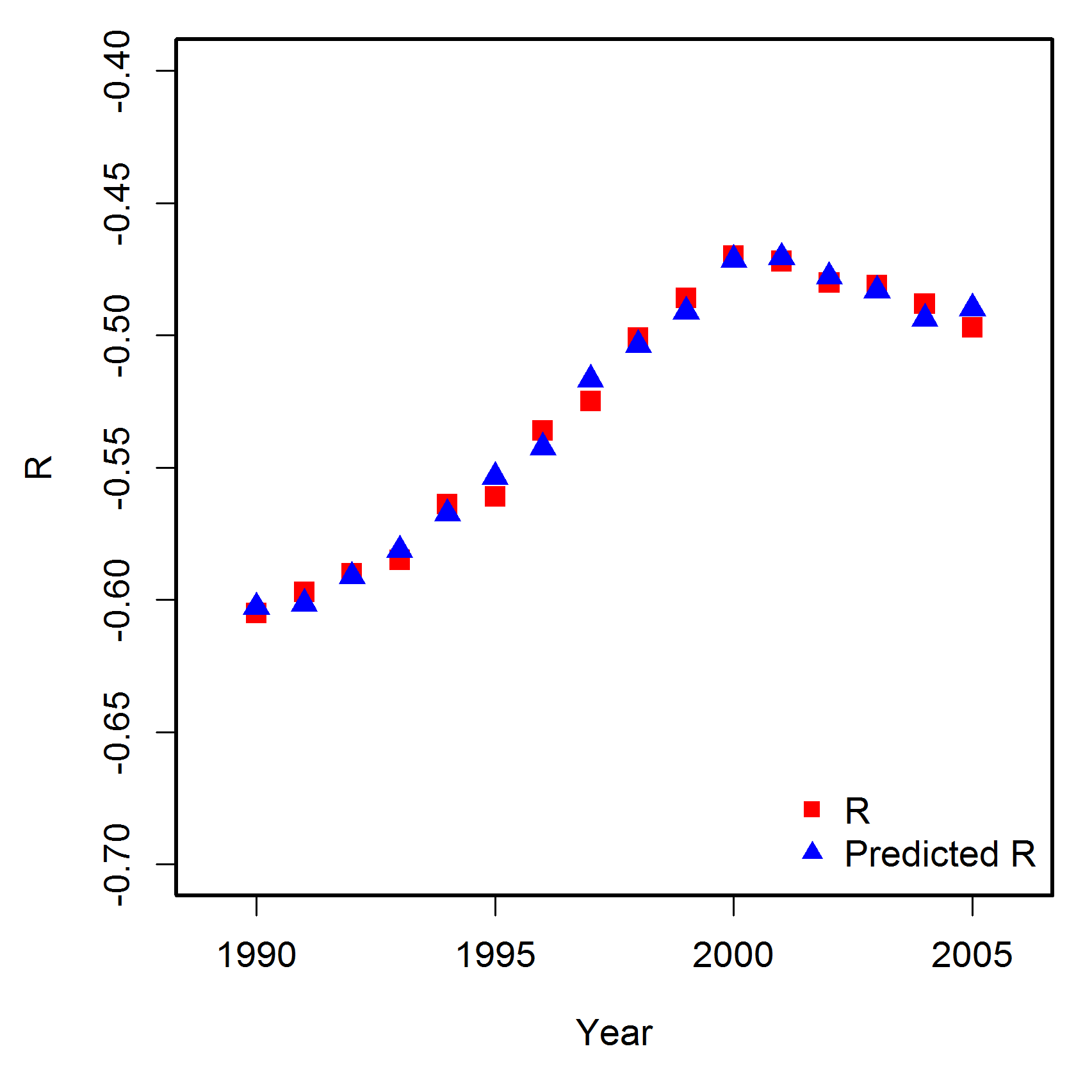


Figure 7. Assessment of the goodness of model fit for the 1990-2005 period with alcohol consumption
